# 4,4,5,5-Tetra­methyl-2-[1,3,6,8-tetra­bromo-7-(4,4,5,5-tetra­methyl-1,3,2-dioxaborolan-2-yl)pyren-2-yl]-1,3,2-dioxaborolane

**DOI:** 10.1107/S1600536812006095

**Published:** 2012-02-17

**Authors:** Ying Chen, Wen-tao Yu, Zhi-qiang Liu, Ping Yu

**Affiliations:** a School of Chemistry and Chemical Engineering, Shandong University, Jinan 250100, People’s Republic of China; b State Key Laboratory of Crystal Materials, Shandong University, Jinan 250100, People’s Republic of China

## Abstract

The complete mol­ecule of the title compound, C_28_H_28_B_2_Br_4_O_4_, is generated by the application of a centre of inversion. In the mol­ecule, the BO_2_ plane is perpendicular to that through the pyrene ring [dihedral angle = 86.27 (13)°]. In the crystal, mol­ecules stack into columns along the *b* axis, the closest contact between these being of the type C—Br⋯π.

## Related literature

For background to the reactions of pyrene, see: Miura & Yamano (1995 ▶). For the structure of the non-brominated derivative, see: Coventry *et al.* (2005 ▶).
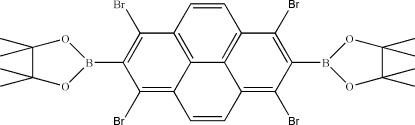


## Experimental

### 

#### Crystal data

C_28_H_28_B_2_Br_4_O_4_
*M* *_r_* = 769.76
Monoclinic, 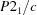

*a* = 15.5047 (10) Å
*b* = 7.5136 (5) Å
*c* = 13.9191 (9) Å
β = 113.961 (1)°
*V* = 1481.78 (17) Å^3^
*Z* = 2
Mo *K*α radiation
μ = 5.46 mm^−1^
*T* = 296 K
0.34 × 0.24 × 0.16 mm

#### Data collection

Bruker APEXII CCD diffractometer
Absorption correction: multi-scan (*SADABS*; Bruker, 2009 ▶) *T*_min_ = 0.258, *T*_max_ = 0.475
8745 measured reflections
3344 independent reflections
2488 reflections with *I* > 2σ(*I*)
*R*_int_ = 0.026

#### Refinement

*R*[*F* ^2^ > 2σ(*F* ^2^)] = 0.035
*wR*(*F* ^2^) = 0.109
*S* = 1.01
3344 reflections
176 parameters
H-atom parameters constrained
Δρ_max_ = 0.60 e Å^−3^
Δρ_min_ = −0.67 e Å^−3^

### 

Data collection: *APEX2* (Bruker, 2009 ▶); cell refinement: *SAINT* (Bruker, 2009 ▶); data reduction: *SAINT*; program(s) used to solve structure: *SHELXS97* (Sheldrick, 2008 ▶); program(s) used to refine structure: *SHELXL97* (Sheldrick, 2008 ▶); molecular graphics: *OLEX-2* (Dolomanov *et al.*, 2009 ▶) and *Mercury* (Macrae *et al.*, 2006 ▶); software used to prepare material for publication: *SHELXL97* (Sheldrick, 2008 ▶).

## Supplementary Material

Crystal structure: contains datablock(s) I, global. DOI: 10.1107/S1600536812006095/tk5049sup1.cif

Structure factors: contains datablock(s) I. DOI: 10.1107/S1600536812006095/tk5049Isup2.hkl

Supplementary material file. DOI: 10.1107/S1600536812006095/tk5049Isup3.cml

Additional supplementary materials:  crystallographic information; 3D view; checkCIF report

## Figures and Tables

**Table 1:** Hydrogen-bond geometry (Å, °) *Cg*1 is the centroid of the C3–C5/C8/C9/C14 benzene ring.

*D*—H⋯*A*	*D*—H	H⋯*A*	*D*⋯*A*	*D*—H⋯*A*
C4—Br2⋯*Cg*1^i^	1.90 (1)	3.48 (1)	4.921 (3)	130 (1)

Symmetry code: (i) 
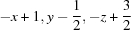
.

**Fig. 1.**
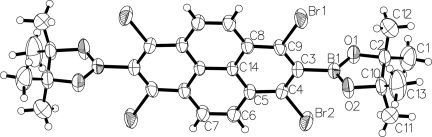
Molecular structure of the title compound. Displacement ellipsoids are drawn at 50% probability level.

**Fig. 2.**
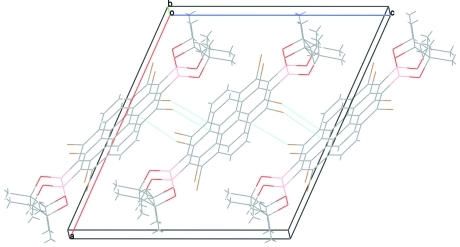
The packing in the title molecules in crystal viewed approximately down the b axis. Intermolecular Br···C (3.414 (3) Å) contacts, being representative of the Br···π contacts are shown as dashed lines.

## supplementary crystallographic information

### Comment

The chemistry of pyrene is strongly position-dependent. For example, in the bromination reaction of 2,7-di-*t*-butylpyrene, the bromide atoms were connected at the 4,5,9,10-positions of pyrene (Miura & Yamano, 1995). However, to our surprise, when the *t*-butyl group is changed to pinacol boronate, the bromination reaction resulted in bromination at the 1,3,6,8-positions as confirmed by the crystal structure described herein.

The molecule, Fig. 1, is centrosymmetric. Before bromination, the two BO_2_ groups are nearly co-planar with the pyrene ring (Coventry *et al.*, 2005). However, they become nearly perpendicular after bromination (dihedral angle 86.27 (13)°).

The molecules pack into columns along the *b* axis, Fig. 2. The most prominent contacts in the structure appear to be of the type C—Br···π, Table 1.

### Experimental

The title compound was synthesized *via* a one-step bromination reaction. The precursor compound, 2,7-di-Bpinpyrene (pin = O_2_C_2_Me_4_) was prepared using the method of Ir-catalyzed borylation (Coventry *et al.*, 2005). To a stirred mixture of 1.36 g (3.0 mmol) of 2,7-di-Bpinpyrene and a small amount of Fe powder (*ca* 0.10 g) in 80 ml of CCl_4_ was added drop-wise a solution of 2.88 g (0.78 mL, 18 mmol) of bromine in 20 ml of CCl_4_ at room temperature. After stirring for 5 h, the mixture was slowly poured into ice water. Then the organic layer was collected and washed with aqueous Na_2_S_2_O_3_ and dried over MgSO_4_. After evaporation, the residue was crystallized from hexane, giving 1.80 g (78% yield) of gray powdered product. Crystals were grown by slow evaporation from its hexane/dichloromethane solution.

### Refinement

Carbon-bound H-atoms were placed in calculated positions [C—H 0.93–0.96 Å, *U*_iso_(H) 1.2–1.5*U*_eq_(C)] and were included in the refinement in the riding model approximation.

### Crystal data

**Table:**

C_28_H_28_B_2_Br_4_O_4_	*F*(000) = 756
*M**_r_* = 769.76	*D*_x_ = 1.725 Mg m^−^^3^
Monoclinic, *P*2_1_/*c*	Mo *K*α radiation, λ = 0.71073 Å
Hall symbol: -P 2ybc	Cell parameters from 3344 reflections
*a* = 15.5047 (10) Å	θ = 2.9–27.4°
*b* = 7.5136 (5) Å	µ = 5.46 mm^−^^1^
*c* = 13.9191 (9) Å	*T* = 296 K
β = 113.961 (1)°	Pod, colourless
*V* = 1481.78 (17) Å^3^	0.34 × 0.24 × 0.16 mm
*Z* = 2	

### Data collection

**Table:**

Bruker APEXII CCD diffractometer	3344 independent reflections
Radiation source: fine-focus sealed tube	2488 reflections with i > 2σ(*I*)
Graphite monochromator	*R*_int_ = 0.026
φ and ω scans	θ_max_ = 27.5°, θ_min_ = 2.9°
Absorption correction: multi-scan (*SADABS*; Bruker, 2009)	*h* = −20→18
*T*_min_ = 0.258, *T*_max_ = 0.475	*k* = −9→9
8745 measured reflections	*l* = −9→18

### Refinement

**Table:**

Refinement on *F*^2^	Primary atom site location: structure-invariant direct methods
Least-squares matrix: full	Secondary atom site location: difference Fourier map
*R*[*F*^2^ > 2σ(*F*^2^)] = 0.035	Hydrogen site location: inferred from neighbouring sites
*wR*(*F*^2^) = 0.109	H-atom parameters constrained
*S* = 1.01	*w* = 1/[σ^2^(*F*_o_^2^) + (0.0653*P*)^2^ + 0.4343*P*] where *P* = (*F*_o_^2^ + 2*F*_c_^2^)/3
3344 reflections	(Δ/σ)_max_ = 0.007
176 parameters	Δρ_max_ = 0.60 e Å^−^^3^
0 restraints	Δρ_min_ = −0.67 e Å^−^^3^

### Special details

**Table:**

Geometry. All e.s.d.'s (except the e.s.d. in the dihedral angle between two l.s. planes) are estimated using the full covariance matrix. The cell e.s.d.'s are taken into account individually in the estimation of e.s.d.'s in distances, angles and torsion angles; correlations between e.s.d.'s in cell parameters are only used when they are defined by crystal symmetry. An approximate (isotropic) treatment of cell e.s.d.'s is used for estimating e.s.d.'s involving l.s. planes.
Refinement. Refinement of *F*^2^ against ALL reflections. The weighted *R*-factor *wR* and goodness of fit *S* are based on *F*^2^, conventional *R*-factors *R* are based on *F*, with *F* set to zero for negative *F*^2^. The threshold expression of *F*^2^ > σ(*F*^2^) is used only for calculating *R*-factors(gt) *etc*. and is not relevant to the choice of reflections for refinement. *R*-factors based on *F*^2^ are statistically about twice as large as those based on *F*, and *R*- factors based on ALL data will be even larger.

### Fractional atomic coordinates and isotropic or equivalent isotropic displacement parameters (Å^2^)

**Table:**

	*x*	*y*	*z*	*U*_iso_*/*U*_eq_	
Br1	0.77662 (3)	0.70726 (6)	0.97202 (4)	0.07199 (18)	
Br2	0.58141 (3)	0.07842 (5)	0.79828 (3)	0.06680 (17)	
O1	0.73711 (17)	0.4128 (3)	0.75325 (18)	0.0521 (6)	
O2	0.81831 (17)	0.2540 (4)	0.89972 (18)	0.0603 (7)	
C1	0.8075 (4)	0.3192 (8)	0.6352 (3)	0.0949 (17)	
H1A	0.7619	0.2248	0.6114	0.142*	
H1B	0.8652	0.2812	0.6316	0.142*	
H1C	0.7835	0.4218	0.5914	0.142*	
C2	0.8258 (2)	0.3653 (5)	0.7461 (3)	0.0537 (9)	
C3	0.6666 (2)	0.4017 (4)	0.8923 (2)	0.0380 (6)	
C4	0.5917 (2)	0.2876 (4)	0.8788 (2)	0.0395 (7)	
C5	0.5247 (2)	0.3199 (4)	0.9201 (2)	0.0377 (6)	
C6	0.4480 (2)	0.2033 (4)	0.9060 (3)	0.0503 (8)	
H6	0.4417	0.0985	0.8681	0.060*	
C7	0.3844 (2)	0.2413 (5)	0.9460 (3)	0.0522 (9)	
H7	0.3348	0.1628	0.9345	0.063*	
C8	0.6087 (2)	0.6003 (4)	0.9941 (2)	0.0404 (7)	
C9	0.6728 (2)	0.5543 (4)	0.9499 (3)	0.0424 (7)	
C10	0.8662 (3)	0.2212 (5)	0.8302 (3)	0.0588 (10)	
C11	0.8343 (5)	0.0358 (6)	0.7828 (6)	0.119 (2)	
H11A	0.8451	−0.0486	0.8382	0.178*	
H11B	0.8697	0.0015	0.7429	0.178*	
H11C	0.7683	0.0387	0.7375	0.178*	
C12	0.8844 (4)	0.5382 (7)	0.7748 (5)	0.0971 (17)	
H12A	0.8460	0.6359	0.7364	0.146*	
H12B	0.9378	0.5261	0.7571	0.146*	
H12C	0.9059	0.5600	0.8489	0.146*	
C13	0.9711 (3)	0.2251 (10)	0.8940 (4)	0.113 (2)	
H13A	0.9882	0.3348	0.9325	0.169*	
H13B	1.0032	0.2160	0.8479	0.169*	
H13C	0.9889	0.1269	0.9423	0.169*	
C14	0.53370 (18)	0.4799 (4)	0.9788 (2)	0.0340 (6)	
B1	0.7425 (2)	0.3542 (5)	0.8473 (3)	0.0397 (7)	

### Atomic displacement parameters (Å^2^)

**Table:**

	*U*^11^	*U*^22^	*U*^33^	*U*^12^	*U*^13^	*U*^23^
Br1	0.0592 (3)	0.0748 (3)	0.1085 (4)	−0.02956 (19)	0.0614 (3)	−0.0371 (2)
Br2	0.0623 (3)	0.0677 (3)	0.0883 (3)	−0.01639 (18)	0.0490 (2)	−0.0388 (2)
O1	0.0482 (13)	0.0696 (15)	0.0472 (13)	0.0173 (11)	0.0281 (11)	0.0108 (11)
O2	0.0525 (14)	0.0954 (19)	0.0458 (13)	0.0283 (13)	0.0331 (12)	0.0213 (13)
C1	0.124 (4)	0.122 (4)	0.058 (3)	0.044 (4)	0.056 (3)	0.016 (3)
C2	0.053 (2)	0.070 (2)	0.0533 (19)	0.0117 (17)	0.0365 (17)	0.0064 (17)
C3	0.0304 (14)	0.0502 (17)	0.0361 (15)	0.0019 (12)	0.0164 (12)	−0.0025 (13)
C4	0.0353 (15)	0.0459 (16)	0.0396 (15)	0.0015 (12)	0.0175 (13)	−0.0087 (13)
C5	0.0303 (14)	0.0468 (16)	0.0389 (15)	−0.0028 (12)	0.0171 (13)	−0.0058 (13)
C6	0.0470 (18)	0.0480 (19)	0.066 (2)	−0.0136 (14)	0.0337 (17)	−0.0207 (16)
C7	0.0449 (18)	0.0520 (19)	0.072 (2)	−0.0169 (15)	0.0358 (18)	−0.0202 (17)
C8	0.0342 (15)	0.0481 (17)	0.0442 (16)	−0.0061 (13)	0.0213 (13)	−0.0086 (13)
C9	0.0312 (15)	0.0519 (18)	0.0493 (18)	−0.0071 (13)	0.0218 (14)	−0.0045 (14)
C10	0.054 (2)	0.073 (2)	0.070 (2)	0.0207 (18)	0.0466 (19)	0.0189 (19)
C11	0.192 (7)	0.055 (3)	0.183 (6)	0.007 (3)	0.153 (6)	0.004 (3)
C12	0.103 (4)	0.078 (3)	0.144 (5)	−0.016 (3)	0.086 (4)	−0.003 (3)
C13	0.057 (3)	0.197 (7)	0.090 (4)	0.045 (4)	0.035 (3)	0.029 (4)
C14	0.0288 (13)	0.0408 (15)	0.0354 (15)	−0.0024 (11)	0.0162 (12)	−0.0050 (12)
B1	0.0338 (17)	0.0473 (19)	0.0427 (19)	0.0011 (14)	0.0204 (15)	−0.0029 (15)

### Geometric parameters (Å, º)

**Table:**

Br1—C9	1.899 (3)	C6—H6	0.9300
Br2—C4	1.899 (3)	C7—C8^i^	1.432 (4)
O1—B1	1.351 (4)	C7—H7	0.9300
O1—C2	1.463 (4)	C8—C9	1.408 (4)
O2—B1	1.336 (4)	C8—C14	1.419 (4)
O2—C10	1.460 (4)	C8—C7^i^	1.432 (4)
C1—C2	1.492 (5)	C10—C13	1.504 (6)
C1—H1A	0.9600	C10—C11	1.535 (7)
C1—H1B	0.9600	C11—H11A	0.9600
C1—H1C	0.9600	C11—H11B	0.9600
C2—C10	1.530 (5)	C11—H11C	0.9600
C2—C12	1.542 (6)	C12—H12A	0.9600
C3—C9	1.380 (4)	C12—H12B	0.9600
C3—C4	1.394 (4)	C12—H12C	0.9600
C3—B1	1.583 (4)	C13—H13A	0.9600
C4—C5	1.397 (4)	C13—H13B	0.9600
C5—C6	1.425 (4)	C13—H13C	0.9600
C5—C14	1.428 (4)	C14—C14^i^	1.426 (5)
C6—C7	1.345 (4)		
			
B1—O1—C2	107.2 (3)	C3—C9—Br1	116.9 (2)
B1—O2—C10	107.7 (2)	C8—C9—Br1	118.9 (2)
C2—C1—H1A	109.5	O2—C10—C13	109.0 (3)
C2—C1—H1B	109.5	O2—C10—C2	103.1 (2)
H1A—C1—H1B	109.5	C13—C10—C2	116.5 (4)
C2—C1—H1C	109.5	O2—C10—C11	106.1 (3)
H1A—C1—H1C	109.5	C13—C10—C11	110.7 (5)
H1B—C1—H1C	109.5	C2—C10—C11	110.7 (4)
O1—C2—C1	109.7 (3)	C10—C11—H11A	109.5
O1—C2—C10	103.0 (2)	C10—C11—H11B	109.5
C1—C2—C10	118.3 (4)	H11A—C11—H11B	109.5
O1—C2—C12	104.4 (3)	C10—C11—H11C	109.5
C1—C2—C12	108.0 (4)	H11A—C11—H11C	109.5
C10—C2—C12	112.5 (4)	H11B—C11—H11C	109.5
C9—C3—C4	116.6 (3)	C2—C12—H12A	109.5
C9—C3—B1	122.0 (3)	C2—C12—H12B	109.5
C4—C3—B1	121.4 (3)	H12A—C12—H12B	109.5
C3—C4—C5	123.7 (3)	C2—C12—H12C	109.5
C3—C4—Br2	117.0 (2)	H12A—C12—H12C	109.5
C5—C4—Br2	119.2 (2)	H12B—C12—H12C	109.5
C4—C5—C6	123.7 (3)	C10—C13—H13A	109.5
C4—C5—C14	117.7 (2)	C10—C13—H13B	109.5
C6—C5—C14	118.6 (2)	H13A—C13—H13B	109.5
C7—C6—C5	121.6 (3)	C10—C13—H13C	109.5
C7—C6—H6	119.2	H13A—C13—H13C	109.5
C5—C6—H6	119.2	H13B—C13—H13C	109.5
C6—C7—C8^i^	121.6 (3)	C8—C14—C14^i^	119.8 (3)
C6—C7—H7	119.2	C8—C14—C5	120.4 (2)
C8^i^—C7—H7	119.2	C14^i^—C14—C5	119.8 (3)
C9—C8—C14	117.3 (3)	O2—B1—O1	114.1 (3)
C9—C8—C7^i^	124.0 (3)	O2—B1—C3	122.8 (3)
C14—C8—C7^i^	118.6 (3)	O1—B1—C3	123.1 (3)
C3—C9—C8	124.2 (3)		

Symmetry code: (i) −*x*+1, −*y*+1, −*z*+2.

### Hydrogen-bond geometry (Å, º)

Cg1 is the centroid of the C3–C5/C8/C9/C14 benzene ring.

**Table:**

*D*—H···*A*	*D*—H	H···*A*	*D*···*A*	*D*—H···*A*
C4—Br2···*Cg*1^ii^	1.90 (1)	3.48 (1)	4.921 (3)	130 (1)

Symmetry code: (ii) −*x*+1, *y*−1/2, −*z*+3/2.
